# Enantiomeric Recognition and Separation by Chiral Nanoparticles

**DOI:** 10.3390/molecules24061007

**Published:** 2019-03-13

**Authors:** Ankur Gogoi, Nirmal Mazumder, Surajit Konwer, Harsh Ranawat, Nai-Tzu Chen, Guan-Yu Zhuo

**Affiliations:** 1 Department of Physics, Jagannath Barooah College, Jorhat, Assam 785001, India; ankurgogoi@gmail.com; 2 Department of Biophysics, School of Life Sciences, Manipal Academy of Higher Education, Manipal, Karnataka 576104, India; nirmaluva@gmail.com (N.M.); harsh.ranawat@outlook.com (H.R.); 3 Department of Chemistry, Dibrugarh University, Dibrugarh, Assam 786004, India; surajitkonwer@dibru.ac.in; 4 Institute of New Drug Development, China Medical University, No. 91, Hsueh-Shih Rd., Taichung 40402, Taiwan; ohnonancy@mail.cmu.edu.tw; 5 Integrative Stem Cell Center, China Medical University Hospital, No. 2, Yude Rd., Taichung 40447, Taiwan

**Keywords:** chirality, racemic mixture, enantiomer, enantiomeric recognition, enantiomeric separation, surface-modified nanoparticle, chiral ligand

## Abstract

Chiral molecules are stereoselective with regard to specific biological functions. Enantiomers differ considerably in their physiological reactions with the human body. Safeguarding the quality and safety of drugs requires an efficient analytical platform by which to selectively probe chiral compounds to ensure the extraction of single enantiomers. Asymmetric synthesis is a mature approach to the production of single enantiomers; however, it is poorly suited to mass production and allows for only specific enantioselective reactions. Furthermore, it is too expensive and time-consuming for the evaluation of therapeutic drugs in the early stages of development. These limitations have prompted the development of surface-modified nanoparticles using amino acids, chiral organic ligands, or functional groups as chiral selectors applicable to a racemic mixture of chiral molecules. The fact that these combinations can be optimized in terms of sensitivity, specificity, and enantioselectivity makes them ideal for enantiomeric recognition and separation. In chiral resolution, molecules bond selectively to particle surfaces according to homochiral interactions, whereupon an enantiopure compound is extracted from the solution through a simple filtration process. In this review article, we discuss the fabrication of chiral nanoparticles and look at the ways their distinctive surface properties have been adopted in enantiomeric recognition and separation.

## 1. Introduction

Chirality is one of those fundamental properties of molecular systems which are ubiquitous in nature [1,2]. Notably, a number of important biological compounds including proteins, amino acids, nucleosides, sugars, and a number of hormones, which are the basic building blocks of life, are chiral and thus the chemistry of many fundamental biological (metabolic and regulatory) processes are directly controlled by such molecular systems [3,4]. Interestingly, enantiomers are mirror images of a chiral compound, e.g., chiral drugs, pesticides, herbicides, etc., which often exhibit profoundly different metabolic, pharmacological, therapeutic and/or toxicological properties [5]. Consequently, the two enantiomers of a chiral compound will react differently with the complementary receptor or enzyme molecule in a biological environment which is highly stereoselective or enantioselective [6]. This issue is particularly important for the pharmaceutical industry where many drugs currently in use are known to be chiral (about 56%) [4] out of which only about 25% are pure enantiomers. Besides, chirality is also important for other industries like agrochemical, food, petroleum, etc. [7,8,9]. For example, approximately 30–40% of the currently registered pesticides, insecticides and herbicides are chiral [6,8,10]. Most of these currently used chiral compounds are racemates or mixtures [11]. Importantly, while one enantiomer of a chiral drug or agrochemical product has desired effect (eutomer), the other may be inactive and/or cause adverse side effects in many cases (distomer) [3,6].

Given the tremendous importance of chirality in many biochemical processes, recent years witnessed enormous efforts among the researchers and manufacturing industries to prepare enantiopure compounds so that an administered chiral compound (drug or other chemical compounds) fits properly to the target binding site or receptor molecule. In this regard, the ideal solution would be the enantioselective synthesis of just one of the enantiomers (synthetic or chiral approach [9,12,13]) by using methods like isolation of natural compounds [14,15], fermentation [16], asymmetric synthesis [17], etc. Notably, the Nobel Prize in Chemistry for 2001 was awarded for the development of catalytic asymmetric synthesis with one half jointly to Knowles and Noyori “for their work on chirally catalyzed hydrogenation reactions” and the other half to Sharpless “for his work on chirally catalyzed oxidation reactions” [18]. Unfortunately very few enantiopure compounds can be obtained from natural sources. On the other hand, the use of asymmetric synthesis is limited due to the high catalyst cost and time-consuming synthesis procedure, albeit it is one of the most powerful methods to produce 100% enantiopure compounds [12,19,20].

**Figure 1:**
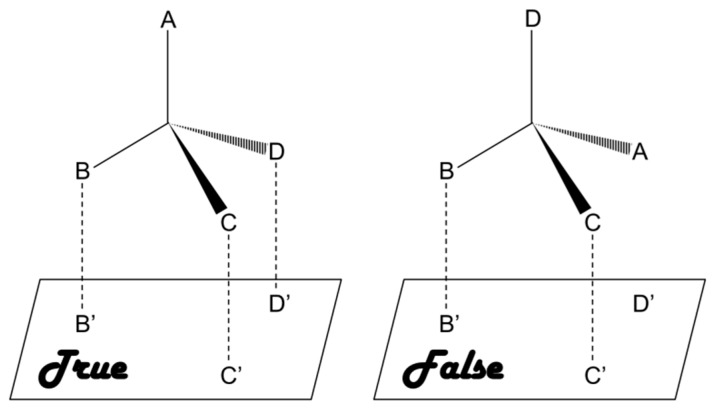
Three-point interaction model for describing the homochiral (true) and heterochiral (false) interactions.

As compared to chiral approach, the racemic approach involves recognition of racemates or mixtures and subsequent separation of the enantiomers, which is relatively cost-effective and presents lower level of difficulty [20]. This approach is based on a three-point interaction between the analyte and a chiral selector [12,21,22,23]. Briefly, a matched pair undergoes a three-point interaction whereas only one or two point interaction takes place in case of a mismatched pair, as shown in Figure 1. A number of chiral selectors, including proteins, cyclodextrin (CD), crown ethers, oligo- and polysaccharides, etc., have been developed till date. Unfortunately there is no universal chiral selector that can be efficiently applied for the recognition and/or separation of all types of chiral compounds [24].

A number of techniques have been developed during the recent past for recognition and/or separation of enantiomers obtained by using the racemic approach [11,25]. Most commonly used methods include chromatographic methods such as high-performance liquid chromatography (HPLC), liquid chromatography (LC), gas chromatography (GC), capillary electrochromatography (CEC), thin-layer chromatography (TLC), micellar chromatography (MC), supercritical fluid chromatography (SFC), and high-speed countercurrent chromatography (HSCCC) [26,27,28,29,30,31,32,33,34,35,36]. Other methods are capillary electrophoresis (CE) [29,30,37], crystallization resolution [38,39], liquid-liquid extraction (LLE) [12,40], membrane separation [41,42,43], kinetic resolution [13], etc. On the other hand, self-disproportionation of enantiomers (SDE) introduced by Soloshonok is another process which efficiently transforms an enantiomerically enriched compound (scalemic mixture, i.e., a mixture of enantiomers at a ratio other than 50:50 or 100:0) into completely racemic (enantio-depleted) and enantiomerically pure (enantioenriched) fractions [44,45,46,47]. Importantly, SDE occurs spontaneously whenever nonracemic compounds are subjected to any physiochemical processes, such as, precipitation, centrifugation, recrystallization, sublimation, force field, achiral chromatography, etc., under totally achiral conditions [48]. 

Each of these methods has their unique abilities for enantiomeric recognition, separation and quantification. Nevertheless, despite the availability of such advanced chiral resolution methods, it is still very difficult to separate the enantiomers with high efficiency because of the intrinsic difficulties associated with these methods, mainly due to the exactly same physiochemical properties of the enantiomer pairs [24,49,50]. In addition, most of them require using chiral columns in place of stationary phase and such processes are often expensive and cumbersome, and do not allow real time analysis. The advantages and limitations of the aforementioned methods that are widely used for chiral resolution are tabulated in Table 1.

Nanoparticles (NPs) represent an entirely new approach to chiral resolution. At present, this generally involves surface modification using chiral ligands; however, recent advances are making the recognition and separation of enantiomers far simpler. One particular achievement in enantiomeric recognition is colorimetric detection, which uses surface-modified NPs to convert recognition events into color changes observable to the naked eye or a UV-Vis spectrometer [52,53,54,55,56,57,58,59,60,61,62]. This makes it ideal for on-site chiral analysis and provides results instantaneously. The working principle is that interactions with specific enantiomers occur on the surface of metal NPs, which means that interactions can be monitored according to changes in surface plasmon resonance (SPR). Furthermore, chiral ligand-capped quantum dots (QDs) have also received considerable attention in this field, due to the size-dependent optical properties, bright chemiluminescence, and excellent chemical stability [63,64,65]. Beside the applications of magnetic nanoparticles (MNPs) such as catalysts, targeted administration and magnetic resonance imaging (MRI) [66,67], researchers have even capped the surfaces of MNPs with chiral ligands to promote specific interactions with the target enantiomers. It may find potential use in the design of new magneto-chiroptical devices [68]. A wide variety of chiral NPs have been developed for enantiomeric recognition, many of which are examined in Section 2. In enantiomeric separation, surface-modified NPs, as chiral selectors, are exposed to a racemic mixture of chiral molecules to perform the selective adsorption of one enantiomer, leaving an excess of the other enantiomer in solution to be removed through multiple rounds of centrifugation. Following centrifugation, the target enantiomers co-precipitate with the chiral NPs, whereas their enantiomeric counterparts remain in the supernatant [52,53,69]. Note that the chiral ligands chosen for enantiomeric separation are susceptible to denaturation and renaturation manipulated by external perturbations such as temperature and magnetic field, making them switchable and reusable. The used nanomaterials and chiral ligands are discussed in Section 3.

## 2. Enantiomeric Recognition by Chiral Nanoparticles

The ability to recognize the molecular chirality of enantiomers is significantly important owing to their critical role in drug development and biochemistry. Current discrimination of enantiomers has remained a challenge due to lack of efficient methods. NP-based enantiomeric recognition and separation have been widely discussed and studied in the past decade. Studies reported chiral -modified nanomaterials for catalysis [70,71], chiral drug separation [71] and sensing [54,55,56,57,58,59,60,63,68,72,73,74]. In this section, we will focus on the enantiomeric recognition and the fabrication of most commonly used gold and silver materials for chiral NPs.

### 2.1. Gold-Based Nanomaterials

Gold-based nanomaterials are the most widely used material for chiral NPs due to its unique optical properties with different shape and size, easy for surface medication and highly biocompatibility [75,76,77]. In addition, gold nanomaterials can be finely tuned by varying the morphological and reaction characteristic to yield desired size and shape of gold nanoparticles (AuNPs) including nanosphere, nanorod, nanocage and nanoflower. The majority of fabrication of gold materials is through gold-sulphur (Au-S) interaction formed between gold surface and either thiol or dithiol group. The surface-assembly monolayer (SAM) of gold-sulphur bond composes a strong and stable linkage for gold materials and the surface-modified moieties such as proteins, antibodies or polymers. Thiol-gold bond fabrication strategy for AuNP was used in several studies [54,58,70,71,73]. Kang et al. [54] developed an electrochemical sensor for enantioselective recognition of 3,4-dihydroxyphenylalanine (DOPA) based on penicillamine-modified AuNPs (Pen-AuNPs). Chiroptical activity was observed in small AuNPs (~2 nm) which are protected by thiolated Pen. This study was based on the chiral interaction between electroactive DOPA as a target molecule and Pen-AuNPs as chiral NPs. Keshvari’s group applied l-cysteine (Cys)-capped AuNPs as a colorimetric sensor for enantioselective detection of naproxen racemic mixtures [73]. The enantioselective and rapid aggregation of l-Cys-modified AuNPs in the presence of *R*-naproxen makes this design capable of visual chiral sensing. The aggregation/agglomeration morphology of AuNPs were successfully demonstrated though transmission electron microscope (TEM), UV-visible spectroscopy, zeta potential, and Fourier-transform infrared spectroscopy (FTIR) measurements.

**Figure 2:**
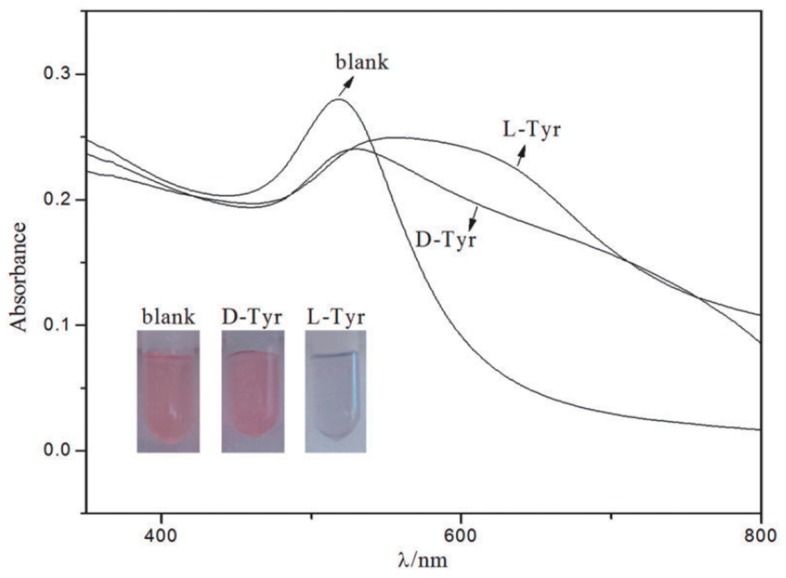
Photographs and UV-vis spectra of EMIML-Tar-AuNPs in the presence of d-Tyr or l-Tyr. Experimental conditions: 1.5 mL EMIML-Tar-AuNPs added with 0.5 mL 1.25 mmol/L d-Tyr or l-Tyr. The figure and the caption have been adapted with permission from [60], Copyright © 2016 OSA.

AuNP bound with chiral molecules on their surface have been used in many studies for enantiomeric recognition. While most commonly employed surface molecules are l-Cys and its derivatives, owing to the ability of the sulfur group easily to bind with NP surface. Other chiral moieties like ionic liquids are now being investigated [78,79]. Ionic liquids are non-molecular ionic solvents where the ions are loosely coordinated, hence their low melting point. They are also called molten or fused salts, with distinct properties such as low vapour pressure, good thermal and chemical stability and fast diffusion and migration of ions. Ionic liquids have been employed successfully in asymmetric synthesis, LC, CE and GC studies. Huang et al. [60] synthesized AuNPs adsorbed with 1-ethyl-3-methylimidazole l-tartrate (EMIML-Tar) and 1-ethyl-3-methylimidazole Lactate (EMIML-Lac) as chiral molecules. EMIML-Tar-AuNPs recognized chiral samples more potently between l-tyrosine (Tyr) and d-Tyr samples, and were considered for the rest of the study. An exclusive red-to-purple color change was observed when l-Tyr was added to the EMIML-Tar-AuNP solution, unlike the D enantiomer. The absorption peaks had also broadened, and spectral differences were quantified using a plot of the extinction ratio (A650/A520) against logarithmic concentration of the Tyr sample solution, as shown in Figure 2. Extinction coefficient values were significantly higher for l-Tyr exposed samples, demonstrating the chiral potency of the EMIML-Tar-AuNP solution. CE analysis was used to characterize the chiral interactions with different amino acids, where it was observed that EMIML-Tar-AuNPs interacted with l-Tyr, tryptophan (Trp) and phenylalanine (Phe) in decreasing order of strength. Visual color changes and absorption spectra changes were confirmed by TEM analysis which showed exclusive aggregation of EMIM-Tar-AuNPs caused by l-Tyr. The proposed mechanism of selective binding was based on shorter distance of the amino group of l-Tyr to carboxyl group of EMIM-Tar-AuNP when comparing the enantiomers of Tyr, and hydroxyl group of Tyr allowing for three-point contact with the NPs when comparing Tyr with Trp and Phe.

When AuNPs are used to discriminate between enantiomer they mostly require surface modification with chiral ligands that enable chiral recognition. Unlike nanospheres, gold nanorods (AuNRs) can take part in enantiomeric recognition independent of surface modifications as they exhibit chirality of their own. They differ from conventional NPs by being slightly elongated in structure and possessing two SPR bands, one for transverse and the other for longitudinal direction of NR. They are also more sensitive to their micro-environment. Therefore, another simple, sensitive, cheap, and easy to operate enantiorecognition method was developed by Wang’s group [58]. Naked AuNR was employed as colorimetric probes for visual recognition of glutamine (Gln) enantiomers. The inherent chirality of AuNRs only aggregated in the presence of d-Gln, thereby resulting in appreciable blue-gray color changes of AuNR solution. On the other hand, no color changes of AuNR solution in the presence of l-Gln. d-Gln also caused a significant decrease in absorbance at 620 nm (longitudinal SPR band). The experiment was also modified and tried to detect enantiomeric excess and was successful in doing so. The experiment was also performed to test the enantiomeric recognition of other amino acids. The results did show some difference for the different enantiomers of these α-amino acids. However, this may be improved by changing the experimental conditions that cater to the specific amino acid being used. Cysteine however showed aggregation of the AuNRs when either of the enantiomers was present. The aggregations could be observed by visualizing the solution using a TEM.

**Figure 3:**
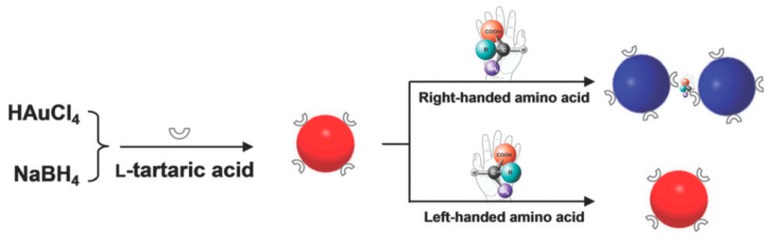
Schematic illustration of visual chiral recognition of right-handed and left-handed amino acid using l-tartaric acid-capped AuNPs as colorimetric probes. The figure and the caption have been adapted with permission from [56], Copyright © 2016 The Royal Society of Chemistry.

Amino acids play a key role in cellular metabolism and protein composition. All amino acids except glycine exhibit chirality. Contemporary techniques of chirality-dependent separation of these amino acids, such as HPLC, CE and GC are expensive and require complicated upstream treatments. For this purpose, Song et al. [56] devised an inexpensive and easy colorimetric probe for visual enantiomeric recognition of right-handed and left-handed amino acid. The synthesis strategy for AuNP was NaBH_4_ reduction method. l-Tartaric acid (TA) was found to be structure-similar to citric acid, which was the commonly used as both a reducing and capping agent for AuNP synthesis. Instead of three carboxyl group (-COOH) as citric acid, l-TA consists of two carboxyl groups, and is adsorbed onto the gold surface through the carboxyl group and provides steric barriers for repulsion. l-TA was also regarded as a chiral selector to separate various enantiomers, including many amino acids. The principle of the proposed technique (Figure 3) is that the change of dispersed AuNP solution to an aggregated state, caused by selective binding of enantiomers, is demonstrated by a red to blue shift. Enantiomer induced calorimetric changes for all 19 α-amino acids at 0.1 mM were observed, whose both L and D forms induced AuNP aggregation. Unlike the other 19 α-amino acids, Cys is the only molecule with a thiol-group, the sulfur atom of which can bind to the surface of AuNP causing the blue shift. On assessing optical rotation of l-TA-capped AuNPs and the amino acids, it was found that all caused a positive rotation, in accordance to the general observation of stronger homochiral bindings relative to heterochiral interactions. Hence, left-handed amino acids, which do not cause aggregation, can be discriminated from right-handed forms. For this, histidine (His) was analyzed further as a sample to prove this model. Solutions of l-His and d-His were analyzed against blank l-TA-capped AuNP solution. A visual change in the color of the solution was observed exclusively in l-His sample, measured as a reduction in the UV-Vis absorption spectra at 520 nm and emergence of a new 700 nm peak. TEM and dynamic light scattering (DLS) measurements established that the aggregation was exclusive to the l-His sample and the d-His sample did not affect the dispersity of l-TA-capped AuNPs in solution. The ratio of absorbance at 700 nm and 520 nm (A700/A520) was studied against different concentrations and enantiomer compositions to enable quantitative analysis. Using this technique for l-His, the limit of detection was 0.015 mM, the binding constant of His enantiomers with l-TA-capped AuNPs being 40.82 and 0.23 of l and d types, respectively. Mechanism of interaction between the right-handed amino acids and l-TA-capped AuNPs was proposed to be through carboxylic, hydroxyl and amino groups by hydrogen bonds. This was supported with FTIR spectral data. Owing to high stability of the synthesized NPs for a considerable amount of time and in a pH range of 3.0–9.0, along with the ability to discriminate enantiomers of 18 amino acids with cheap spectrophotometers as the chief measurement device, this technique can potentially be used for high-throughput applications.

**Figure 4:**
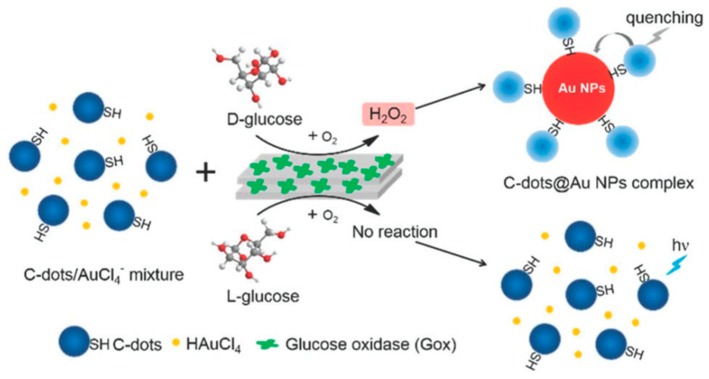
Schematic representation for the generation of C-dots@AuNP complex trigged by the stereoselective enzymatic reaction for the discrimination of glucose enantiomers. The figure and the caption have been adapted with permission from [57], Copyright © 2018 The Royal Society of Chemistry.

Additionally, Zhou et al. [57] established a carbon dots-gold nanoparticle (C-dots@AuNP) complex for chiral discrimination of glucose enantiomers according to colorimetric and fluorescence dual-mode signals. Cysteine was selected as a precursor to generate sulfhydryl decorated-C-dots, which is responsible for the formation of the C-dots@AuNP complex based on the strong tendency of the sulphur element to conjugate onto the surface of AuNPs. H_2_O_2_ is produced as a result of the enzymatic action of glucose oxidase (Gox) and this by-product helps formation of AuNPs by facilitating the reduction chloroauric acid. As shown in Figure 4, in the presence of carbon dots decorated with sulfhydryl groups (derived from Cys), the AuNPs form a complex—C-dots@AuNP. The carbon dots used show an absorption peak at around 350 nm and at an excitation wavelength of 340 nm, they fluoresce with maximum emission at 424 nm. The intensity of this emission is quenched due to the AuNPs when the C-dots@AuNPs complexes are formed. Thus fluorescence spectra in this case can be used to tell d- and l-glucose apart. Such a method is much simpler and faster when compared to using chiral ligands to modify the surface of NPs. The difference can also be seen visually since in the presence of d-glucose the solution turns reddish while it remains colorless when l-glucose is used. TEM images of glucose oxidase catalytic reaction solution show that C-dots cluster around AuNPs when d-glucose is used whereas no AuNPs are formed in the first place when l-glucose is used and C-dots are seen in a monodisperse state.

### 2.2. Silver-Based Nanomaterials

**Figure 5:**
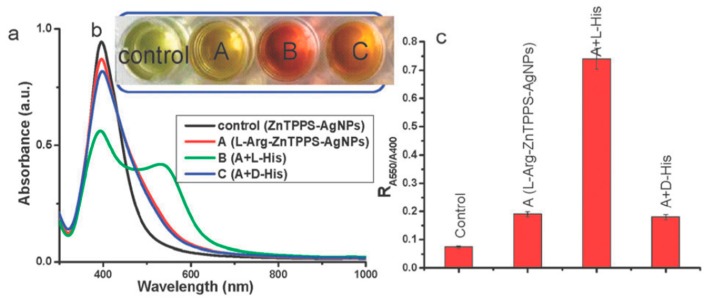
Schematic representation The UV-vis spectra (**a**), photographic images (**b**) and the value of R (A550/A400) (**c**) of silver nanoparticle cluster solution in the presence of d- or l-His. It is operated as follows: 0.5 mL of 1 mM solution of d- or l-His was added to 1.5 mL l-Arg-ZnTPPS-AgNPs solution and mixed for 10 min before measuring, respectively. The figure and the caption have been adapted with permission from [55], Copyright © 2012 The Royal Society of Chemistry.

Silver-based nanomaterials are other widely used materials for enantiomeric recognition, separation and sensing. Silver nanomaterials possess unique chemical and physical properties like higher extinction coefficient, excellent biocompatibility and strong SPR absorption. This phenomenon depends on the size, shape and inter-particle distances of metal NPs and the surrounding environment. In addition, similar to AuNPs, AgNPs were found to have the inherent chirality. Those properties make AgNPs suitable for the fabrication of chiral sensors. Sun’s group designed a sulfonated-substituted zinc tetraphenylporphyrin (ZnTPPS)-modified AgNP as colorimetric sensor for chiral detection of l-arginine (Arg) and His [55]. ZnTPPS was selected to fabricate the AgNPs for not only providing stability of NPs but also introducing enantiomeric recognition for l-Arg and l-His via Zn binding. ZnTPPS-AgNPs were found to be coordinately bound to N-H of l-Arg leading to formation of AgNP clusters (l-Arg-ZnTPPS-AgNPs) which were then characterized by TEM and UV-vis spectroscopy. In the presence of l-Arg, a color change from light yellow to yellow can be observed accompanied by a decrease in absorbance at 400 nm that arises from the SPR absorption of dispersed AgNPs. l-Arg induces aggregation of the ZnTPPS-AgNPs to form AgNP clusters, l-Arg-ZnTPPS-AgNPs. This was confirmed by TEM. Methionine (Met), His, Phe, Tyr and glutamic acid were tested using these modified AgNPs but only Met and His showed an obvious color change and His was chosen for further investigation. It was found that only l-His induced a color change from yellow brown to red accompanied by a significant decrease in the absorbance at 400 nm (Figure 5). A new band at 550 nm was observed and the absorbance ratio (R) at the two wavelengths were determined and was found to be enhanced only in the presence of l-His. d-His on the other hand had none of these effects, thereby proving the good chiral selectivity of these AgNPs. l-His induced further aggregation of the modified AgNPs and caused them to form larger agglomerates and this was clearly seen using TEM. In circular dichroism spectra, these NPs showed a slight chiral signal in the presence of d-His whereas l-His had a much stronger one. To satisfy the three-point contact model for enantiomeric recognition, they proposed a possible interaction mode between l-His and the AgNPs clusters via the carboxylic, amino and imidazole groups through electrostatic and hydrogen bond interactions. In a quantitative analysis study, they found that at very low concentrations of His, the NPs showed low chiral selectivity. Hence it showed in the end that this method was effective for enantiomeric recognition of His and Met.

Marzieh et al. [61] used chitosan-capped silver nanoparticles (CS-AgNPs) for enantiomeric recognition of the essential amino acid, Trp. They scanned optical cells containing the NPs and d- or l-Trp and the color values of each optical cell were then analyzed. The NPs were characterized using FTIR spectra, TEM, X-ray diffractometer (XRD) and UV-vis spectroscopy after their synthesis. When given concentrations of the Trp enantiomers were added separately to a solution of CS-AgNPs, UV-vis spectra were recorded after 30 min. A control without Trp was also tested in the same manner. While scanning for the color of the solution at selected areas of the scanned image for RGB analysis, the values were averaged. MATLAB software was used for assessing different types of colors and obtaining the CMYK values. Differences between CMYK values of the blank control were compared to those of solutions containing either of the Trp enantiomers. The maximum absorbance of the AgNPs was observed at 404 nm and represented the characteristic SPR band of the AgNPs. TEM images of the CS-AgNPs showed that they were uniformly distributed in aqueous solution with an average diameter of about 15 nm and their concentration was calculated using Beer’s law. In the presence of l-Trp at sufficiently detectable concentrations, a color change from a yellowish to brown was observed and the CS-AgNPs were found to be aggregated when observed using TEM. No such observations were detected when d-Trp was used. Experiments were also performed by varying various experimental conditions to find the optimal settings of the experiment. They also found that enantiomeric composition of Trp could be determined from corresponding scanometry and spectrophotometric linear calibration curves. The possible interaction mechanism between the CS-AgNPs and l-Trp was proposed to be the hydrogen bond formation between -OH groups of surface of CS-AgNPs and the amine and carboxyl groups of l-Trp.

**Figure 6:**
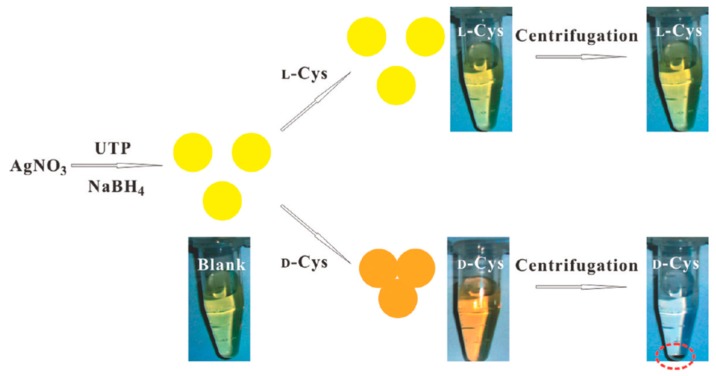
Colorimetric Discrimination of l- and d-Cys Using UTP-Capped AgNPs. The figure and the caption have been adapted with permission from [52], Copyright © 2011 American Chemical Society.

AgNPs exhibit a distance-relevant color and higher extinction coefficient than that of AuNPs of the same size, thereby making them more reliable as color reporting agents for colorimetric sensor design. Zhang et al. [52] used uridine 5′-trisphosphate (UTP)-capped AgNPs for chiral detection of Cys enantiomers. It was found that in the presence of d-Cys an appreciable color change from yellow to red took place but l-Cys showed no such change, as shown in Figure 6. Moreover, when these UTP-capped AgNPs were added to a racemic solution of Cys, they interacted specifically with one enantiomer leaving an excess of the other after centrifugation thus enabling enantiomeric separation. When UV-vis spectroscopy was performed, the peak at 400 nm was seen to undergo a red shift to 520 nm in the presence of d-Cys due to the SPR of AgNPs. Aggregation of the NPs also took place resulting in the decrease of the SPR band at 400 nm. This band was consequently broadened around 450-600 nm. No such effects were reported when l-Cys was used. The absorbance ratio at 520 nm and 400 nm relates to the quantities of dispersed and aggregated AgNPs and thus this was chosen to evaluate the performance of the AgNPs. 

This ratio resulted in significant differences when d- and l-Cys were used. The ratio was found to increase with increasing concentration of d-Cys from 0.1 to 100 micromolars. No such event took place in case of l-Cys except when its concentration was above 100 micromolars where it may cause the AgNPs to lose stability and aggregate, but data suggested that aggregation caused by d-Cys was much more sensitive by at least two times that of l-Cys. UTP analogs were also tested in their role as stabilizing agents on the surface of the AgNPs. When adenosine 5′-triphosphate (ATP) was used, there was negligible discrimination between the two enantiomeric forms of Cys but gradual decrease in the 400 nm and increase in the 520 nm was observed along with the same color change as before. This proved that ATP coated AgNPs could be used to quantitatively detect Cys but not discriminate between the enantiomeric forms. Furthermore, Tashkhourian et al. [62] established AgNP-based colorimetric sensor to discriminate *R*-citalopram (Cit). Citalopram is a selective serotonin reuptake inhibitor used as an antidepressant drug. *S*-Cit was found 150 more potent than racemic Cit or *R*-Cit. Therefore, a simple and reliable detection system for chiral Cit is ponderable in pharmaceutical field.

Gold and silver nanoparticle-based enantiomeric recognition have been widely discussed and studied in the past decade. However, Bruckner et al. [80] demonstrated the use of polysaccharide-ester based amino acid enantiomers using gas chromatography-mass spectrometry (GCMS) in analysis of 24 h urine and blood sera. The experimental procedure involved GC of *N(O)*-pentafluoropropionyl (PFP)-amino acid-(2)-propyl esters on *N*-propionyl-l-valine-*tert*-butylamide polysiloxane, and subsequent filtration by selected ion monitoring mass spectrometry (SIM-MS). The PFP-(2)-propyl esters were synthesized using a mixture of 14 d,l-amino acids and γ-aminobutyric acid (Gaba). The highest amounts of chiral amino acids were determined as d-Serine and d-Alanine, both in urine samples and blood sera, with blood sera in relatively much lower amounts. Samples from fasting patients revealed decreased levels of d-amino acids, while time dependent analysis showed a continuous excretion in the urine.

## 3. Enantiomeric Separation by Chiral Nanoparticles

Applications of NPs in enantiomeric separation have shown promising results by enhancing the resolution, processing time and efficiency of the conventional separation methods [81]. The use of NPs in enantiomeric separation dates back to 1989 when Wallingford and Ewing reported the first application of polymer NPs (mentioned as monomolecular particles) in CE [82,83,84]. Since then a varieties of NPs have been used for efficient separation of enantiomers. Table 2 summarizes some of the recent applications of nanostructures in enantioseparation science.

Notably, NPs represent a state of matter in the transition region between solids and molecular structures in the size range of 1~100 nm [103], where charge carriers are confined in one, two or all of the three dimensions resulting in quantum well structures (nanosheets), quantum wire structures (nanowires) or quantum dot structures (nanoparticles), respectively. Due to quantum confinement and large surface-to-volume ratio (*s*/*v* > 1), such structures exhibit unique size-dependent optical, electronic, magnetic, and catalytic properties [120]. In addition, nanostructures possess better chemical stability, low cytotoxicity, significant mechanical strength, and are easily modifiable in terms of their size, shape and surface properties [91,103]. Importantly, NPs enhance the performance of enantioseparation process due to their extremely large *s*/*v* ratio (e.g., nanospheres of 10 nm diameter can have a surface area as large as 600 m^2^/cm^3^ [121]) that maximizes the amount of binding sites [69,81]. In the following sections we summarize some of the recent applications of different types of nanostructures for the separation of enantiomers.

### 3.1. Metal Nanoparticles

Recent years have witnessed tremendous applications of metal NPs in diverse fields of research including material science, biotechnology, biomedical engineering, targeted drug delivery, environmental, etc. [122,123,124]. In addition, the potential of metal NPs in separation science has been already well recognized due to their easy synthesis procedure, large *s*/*v* ratio, controllable particle size, narrow size distribution, extraordinary biocompatibility, molecular detection properties, etc. [24,89,125].

In a pioneering work in 2005, Choi et al. [87] employed sulfonated β-CD as a chiral selector and Ag colloids as an additive in CE for the enantiomeric separation of arylalcohols (1-phenyl-1-propanol, 1-phenyl-2-propanol, and 2-phenyl-1-propanol). The group observed that the addition of Ag colloid to the running buffer improves the resolution significantly. Another simple, reliable and highly efficient colorimetric platform for enantiomeric separation and detection was proposed by Zhang et al. [52] by using UTP nucleotide-capped AgNPs. The enantioselective aggregation of AgNPs allowed the group achieving rapid colorimetric separation of d- and l-Cys.

**Figure 7:**
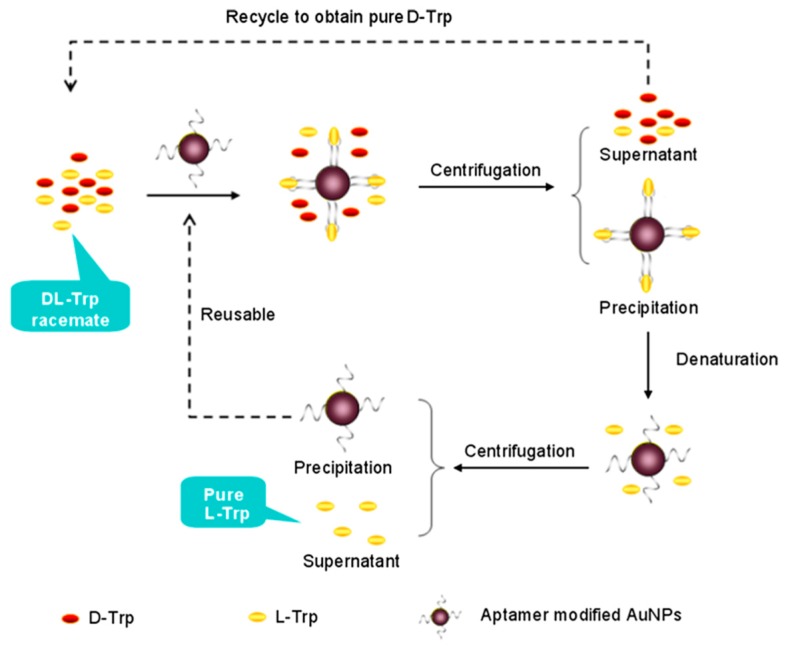
The schematic of the enantioseparation of d,l-Trp based on functional nucleic acids modified Au nanoparticles. The figure and the caption have been adapted with permission from [69], Copyright © 2013 Wiley.

AuNPs have also been applied extensively for enantiomeric separation. In 2005, Shao et al. [126] synthesized human serum albumin (HSA) immobilized gold nanotube membranes for the separation of warfarin enantiomers. Li et al. [127] used bovine serum albumin-gold nanoparticles (BSA-AuNPs) conjugate as chiral stationary phases (CSPs) in open-tubular capillary electrochromatography (OTCEC) for the enantiomeric separation of ephedrine and norephedrine isomers. Good resolutions of 1.18 and 2.15 were obtained for norephedrine and ephedrine isomers, respectively, within 250 s at an effective separation channel length of 36 mm. In another work, Yang et al. [90] employed thiolated β-CD-modified AuNPs as chiral selector in pseudostationary phase-CEC to separate four pairs of dinitrophenyl-labeled amino acid enantiomers and three pairs of drug enantiomers with enantioseparation resolution up to 4.7. The repeatability of this method has been improvised in a later work by immobilizing the thiolated β-CD-modified AuNPs onto the inner wall of a capillary to serve as stationary phase for enantioselective OTCEC separation [128]. Interestingly, Shukla et al. [88] reported a unique strategy for enantioselective adsorption of propylene oxide (PO) by using either d- or l-Cys-modified AuNPs. In particular, the group demonstrated the ability l-Cys (d-Cys)-modified AuNPs to selectively adsorb the (*R*)-propylene oxide ((*S*)-propylene oxide). Notably, this work was extended by using d- or l-Cys to functionalize tetrahexahedral (THH, 24-sided) AuNPs to separate a real chiral pharmaceutical-propranolol [89]. Recently, nucleic acid aptamer-functionalized AuNPs were used as chiral selector for the separation of racemic d,l-Trp. The method used centrifugation to separate the precipitate formed by the aptamer-specific enantiomer (l-Trp) bounded AuNPs (Figure 7) [69]. In another work, Su et al. [53] demonstrated the potential of laboratory synthesized chiral *N*-acetyl-l-Cys-capped AuNPs for high throughput enantiomeric separation of amino acid enantiomers via centrifugation. Recently, monolayer and multilayer AuNPs film capillary columns were fabricated through layer-by-layer self-assembly of AuNPs and their subsequent functionalization through self-assembly of thiolated β-CD in OTCEC [129]. It was observed that, as compared to monolayer AuNPs film capillary column, the three layer AuNPs film capillary column possess superior enantioseparation performance. Further, in 2018, Liu et al. [86] successfully added streptomycin-modified AuNPs in background electrolyte (BGE) solution in CE for the first time to separate a number of drug racemates, such as, adrenaline hydrochloride, noradrenaline bitartrate, and isoprenaline hydrochloride.

### 3.2. Metal Oxide Nanoparticles

**Figure 8:**
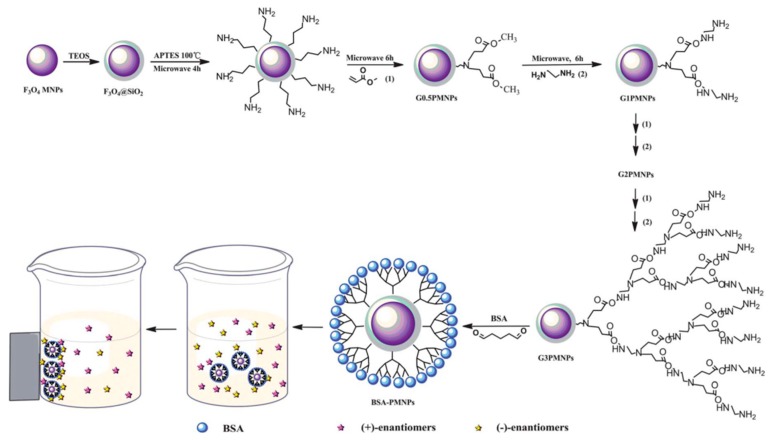
Preparation of BSA–PMNPs and the direct separation of the racemates. The figure and the caption have been adapted with permission from [66], Copyright © 2013 The Royal Society of Chemistry.

In recent years, metal oxide nanoparticles such as magnetite (Fe_3_O_4_), titania (TiO_2_), Zirconia (ZrO_2_), etc., have become crucial in enantiomeric separations, catalysis, sensing devices, cell labeling, drug delivery, and biomedical applications [101,130,131,132]. Notably, nanoscale Fe_3_O_4_ is one of the important phases of iron oxide and has been extensively used in the field of enantiomeric separation due to the possibility of their easy manipulation even at a lower magnetic field [85,133]. In a seminal work, Choi et al. [91] prepared magnetic silica nanoparticles, MSNPs (spherical SiO_2_ NPs containing Fe_3_O_4_) of average size 300 nm in order to separate enantiomers of *N*-(3,5-dinitrobenzoyl)-α-amino acid *N*-propylamides. Interestingly, the group named the enantioseparation process as “enantioselective fishing” since the MSNPs tagged with an appropriate chiral selector readily formed complexes with one of the enantiomers which was subsequently separated simply by using a magnet. Enantiomeric separation of amino acids were also performed by employing water-soluble β-CD-modified Fe_3_O_4_ NPs fabricated by using a simple and convenient chemical route [134]. The group cleverly utilized a strategy to chirally select amino acids by β-CD and subsequently use Fe_3_O_4_ NPs as magnetic separators. In another work, enantiomeric separation of aromatic amino acids (d,l-Trp, Phe and Tyr) has also been achieved by using carboxymethyl-β-cyclodextrin (CM-β-CD)-functionalized MSNPs [92]. In the meantime, Arslan et al. [132] developed a novel enantioselective sorption approach based on Fe_3_O_4_ NPs functionalized with three different β-CDs. The group established that the novel Cd-grafted Fe_3_O_4_ NPs significantly enhance their enantioseparation capabilities towards a set of chiral carboxylic acid molecules. Notably, Sayin et al. [135] grafted *p-tert*-butylcalix[8]arene derivative (C[8]-C4-COOH) onto Fe_3_O_4_ NPs and used both C[8]-C4-COOH and Fe_3_O_4_ NPs for the encapsulation of lipase in order to achieve enhanced catalysis and enantioselective resolution of racemic naproxen methyl ester. Chiral MNPs based on BSA and Fe_3_O_4_ NPs have been used recently to separate chiral drugs (ibuprofen and ofloxacin) [93] and amino acids (Figure 8) [66]. Most recently, chiral core/shell structured MNPs were fabricated by using poly(*N*-isopropylacrylamide-co-glycidyl methacrylate)-β-cyclodextrin (PNG-CD) to form smart polymer brushes (shell) onto polydopamine coated Fe_3_O_4_ MNPs (core) [136]. The smart NPs (Fe_3_O_4_@PDA@PNG-CD), fabricated in this work, showed excellent magnetic separability where the β-CD units acted efficiently as the chiral selector to selectively recognize and bind the desired enantiomer forming host–guest inclusion complexes.

On the other hand, TiO_2_ and ZrO_2_, have also been well recognized as a stationary phase due to their favorable physiochemical properties [137,138,139,140]. The applicability of TiO_2_ NPs in enantiomeric separation has been demonstrated by first coating it onto the micron sized silica spheres [94]. The TiO_2_/SiO_2_ particles were further coated with cellulose tris-(3,5-dimethylphenylcarbamate) (CDMPC) to prepare the CSP for the separation of eight indole ring derivative enantiomers. Notably, the use of TiO_2_ NPs as additive in CE for the simultaneous separation of eight β-adrenergic drugs has also been reported [96]. In another work, a ZrO_2_ based HPLC packing material ZrO_2_/SiO_2_ was prepared by multilayer coating of ZrO_2_ NPs on the surfaces of silica spheres using layer-by-layer self-assembly technique [141]. The group analyzed the potential of ZrO_2_/SiO_2_ material for enantioseparation applications. Moreover, in another work, Kumar et al. [96] reported the fabrication of Fe_3_O_4_@ZrO_2_ microspheres (Fe_3_O_4_ magnetic core covered by a ZrO_2_ shell) of average size 340 nm, functionalized with CDMPC, for the separation of racemic chiral drugs. Interestingly, the excellent recyclability of the synthesized chiral ZrO_2_ magnetic microspheres is quite promising for their use in the multiple enantiomeric separations.

### 3.3. Carbon-Based Nanomaterials

Importantly, among different varieties nano-engineered materials used for enantiomeric separation, carbon nanostructures have also attracted much attention due to their high mechanical strength, good chemical stability, high elasticity, and high thermal conductivity [142,143]. So far, a number of allotropic carbonaceous nanomaterials, such as, carbon nanotubes (CNTs), graphene and graphene oxides (GO) have been successfully applied in this area.

#### 3.3.1. Carbon Nanotubes

Notably, CNTs can be considered as graphite sheets (sp^2^ hybridised carbon atoms) wrapped up in the form of a cylinder that are usually capped by a fullerene-like structure with diameters ranging from few Å [144,145,146] to tens of a nanometer [102,147] and length up to a few micrometer (may extend to centimetres in certain occasions) [103,148,149]. CNTs were first discovered by Iijima [150] and have been one of the most researched materials of 21st century [151]. CNTs can be broadly classified into two types: multi-walled carbon nanotubes (MWCNTs) comprised of more than one coaxially rolled up graphite sheets and single-walled carbon nanotubes (SWCNTs). Due to their unique physicochemical properties and large chemically active surface area, which have been thoroughly discussed in several review articles [152,153,154,155], both MWCNTS and SWCNTs are emerging as one of the most promising candidates for numerous potential applications [156,157,158]. In the context of enantiomeric separation, CNTs are capable of improving the speed, selectivity, stability, and efficiency in chiral chromatographic separation [24,159]. In an exciting work, Ahmed et al. [100] demonstrated the creation of a CSP with good enantioselectivity by encapsulating small amount of SWCNTs into polymer monolithic backbones. Using enantioselective nano-HPLC separation, the group successfully separated twelve classes of pharmaceutical racemates. Under optimum concentration conditions, the group achieved satisfactory repeatability and observed that (6,5) SWCNTs displayed higher enantioselectivity as compared to the (7,6) SWCNTs. Notwithstanding the enormous potentials of CNTs to be used as sorptive material for enantiomeric separation, there are very few reports where such non-functionalized CNTs have been used for the separation of chiral compounds through affinity chromatography. This might be due to the reason that CNTs, especially chiral CNTs, alone may not be an effective adsorbent for enantiomeric separation [160,161].

**Figure 9:**
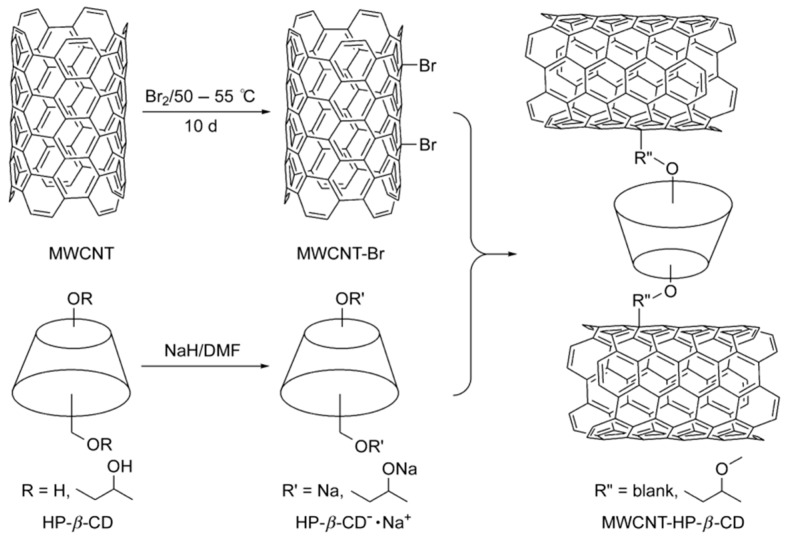
Schematic presentation of preparation of HP-β-CD modified MWCNTs. The figure and the caption have been adapted with permission from [102], Copyright © 2011 Wiley.

Nevertheless under strong and specific chemical conditions the surfaces CNTs can be modified/functionalized to enhance their solubility and other intrinsic properties which render them CSPs or pseudostationary phases [24,97]. There are a number of efforts in the recent past where enantiomeric separation was successfully performed by the modification of CNTs with chiral selectors such as β-CD. For example, Na et al. [101] demonstrated a new strategy for enantiomeric separation of clenbuterol by CE using modified CNT as chiral selector in 2004. The group reported the formation of a large surface area platform by the NPs modified with β-CD that served as a pseudostationary chiral phase for the enhanced separation of enantiomers. In a similar work to separate the same kind of enantiomers, Yu et al. [102] reported a method to functionalize MWCNTs with hydroxypropyl-β-cyclodextrin (HP-β-CD) in order to use as a stationary phase additive in TLC, as shown in Figure 9. Recently, carbogenic NPs, carboxylated SWCNTs and carboxylated MWCNTs were used to modify enantioseparation systems to form pseudostationary phases with dextrin as chiral selectors in electrokinetic chromatography (EKC) [104]. Particularly the group established that the introduction of NPs into the buffer significantly could enhance the resolution of several drug enantiomers namely sulconazole, ketoconazole, citalopram hydrobromide, and nefopam hydrochloride. Under optimized dextrin concentration, buffer pH and buffer concentration resolution time of 15 min with resolution values in the range 1.41–4.52 were achieved in this work. In another work, a novel chiral selector was developed by conjugating BSA which is a type of protein with carboxylic SWCNTs [98]. The efficient enantioseparation ability of the BSA-SWCNT conjugate was illustrated by the successful separation of Trp enantiomers within 70 s with a resolution factor of 1.35 and separation length of 32 mm obtained by using poly(methyl methacrylate) based microchip electrophoresis. The potential of concentrated surfactant-coated MWCNTs as an alternative to other chiral selectors like CDs were also successfully demonstrated by using partial filling of the capillary in EKC at different experimental conditions such as pH, addition of organic modifiers, potential and injection time [97].

Recently, Zhao et al. [162] allowed SWCNTs to bind with the inner wall of a capillary column in GC in order to enhance the enantiomeric separation by achieving larger surface for the chiral ionic liquid stationary phase, which in turn increased the interaction between the stationary phases and the analytes. They prepared two capillary columns for the chromatography process: column A containing only the chiral ionic liquid and column B containing a mixture of the chiral ionic liquid and SWCNTs. Interestingly out of 12 model racemates, column B successfully separated eight chiral compounds as compared to the column A which separated only four of such compounds. In another work, Guillaume et al. [99] successfully developed a simple approach by using HPLC for the enantiomeric separation of amino acids by functionalizing SWCNTs with pyrenyl derivative of a chiral aminoglycoside called neomycin A (PNA). Magnetization of CNTs also showed promising results in the separation of enantiomers. Tarigh et al. [163] used the chiral selector l-threonine (Thr), anchored to the surface of magnetic MWCNTs, to successfully separate d,l-mandelic acid within a time period of 10 min.

#### 3.3.2. Graphene and Graphene Oxide Nanomaterials

Graphene is, on the other hand, a two-dimensional (2D) sheet of sp^2^ hybridized carbon atoms with single atom thickness. In principle, graphene can be regarded as the basic building block of fullerenes (0D structure), carbon nanotubes (1D structure) and graphite (3D structure) [164,165]. It is the thinnest yet the strongest known material which exhibit exceptional optical, electronic, thermal, and adsorptive properties [166,167]. The profound impact of graphene in the field of material science has been well recognized by the Nobel Prize in Physics for 2010 awarded jointly to Geim and Novoselov of The University of Manchester, UK for their groundbreaking experiments related to graphene [168]. On the other hand, oxidization of graphite leads to the formation of graphene oxide (GO) decorated with various oxygen containing functionalities such as epoxide, carbonyl, carboxyl, and hydroxyl groups [169]. GO is attracting enormous attention due to its easy synthesis procedure, high yield and satisfactory dispersibility in organic solvents [170].

Notably, graphene and GO exhibit considerable potential in separation science [165] and has been used by several research groups for the enantiomeric separation of chiral molecules. For instance, Tu et al. [106] reported the graphene assisted resolution of two racemic drugs propranolol and ofloxacin using pure d-(–)-TA as the chiral selector in TLC. Interestingly, computational simulations using density functional theory also showed the applicability of nanoporous graphene, when functionalized by a chiral bouncer molecule [171]. In another exciting work, Candelaria et al. [105] demonstrated the fabrication of new type of chemically stable, versatile and cost-effective graphene-based CSPs for enantiomeric separation using LC. The group employed functionalized mesoporous 3D graphene nanosheets for the enantiomeric separation of pharmaceutical grade racemic mixtures of model ibuprofen and thalidomide.

**Figure 10:**
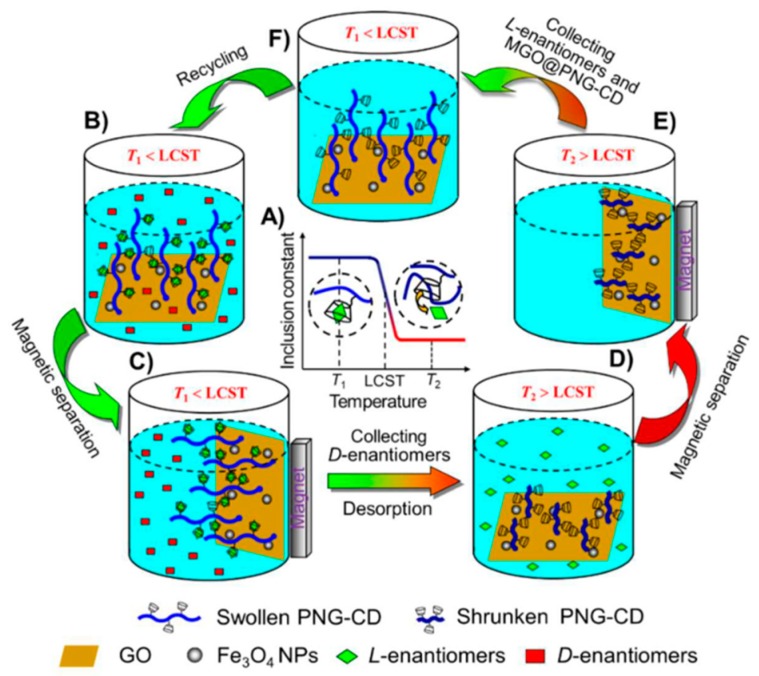
Schematic illustration of the thermosensitive chiral recognition and enantioseparation of AAs enantiomers by MGO@PNG-CD. (A) As the temperature is below the LCST of PNIPAM chains (T1), the β-CD units in PNG-CD can selectively recognize and accommodate l-enantiomers to form stable host-guest inclusion complexes, while as the temperature is above the LCST of PNIPAM chains (T2), the loaded l-enantiomers can desorb from the β-CD cavities automatically because of the reduction in the inclusion constants of β-CD/l-enantiomers complexes; (B) The MGO@PNG-CD is added in the AAs enantiomeric solution at T1 and the PNIPAM chains are swollen, and then l-enantiomers are recognized by β-CD; (C) Through an external magnetic field l-enantiomers are loaded on the MGO@PNG-CD, and d-enantiomers are remained in the enantiomeric solution for subsequent separation; (D) while the operating temperature is above the LCST of PNIPAM (T2), the PNIPAM chains occur to collapse, and the loaded l-enantiomers are released, and the MGO@PNG-CD is recycled; (E and F) the regenerated MGO@PNG-CD is recovered using a magnet and reused. The figure and the caption have been adapted with permission from [111], Copyright © 2018 American Chemical Society.

In case of GO, Liang and his coworkers, in a series of publications, employed OTCEC for the separation of different types of enantiomers by using β-CD conjugated GO-magnetic nanocomposites (GO/Fe_3_O_4_ NCs) [108], BSA-conjugated GO-magnetic nanocomposites (GO/Fe_3_O_4_) [172] and BSA conjugated polydopamine-GO nanocomposites (PDA/GO/BSA) [109] as the stationary phases. Exploiting the large surface area, excellent biocompatibility of graphene and rough surface morphology of GO, the group used the OTCEC microdevices to successfully separate Trp, Thr and propranolol enantiomers with reasonably good resolution factors. Notably, as compared to other similar techniques, the group reported better resolution factor for the enantiomeric separation of d,l-Trp by using the novel PDA/GO/BSA stationary phase. Li et al. [170] reported a facile and efficient strategy to chirally functionalize GO with optically active helical polyacetylene chains. The group successfully used the GO hybrid as a chiral inducer for the enantioselective crystallization of alanine enantiomers where l-alanine was induced to crystallize in the form of rodlike crystals. There are also examples where GO coated fused silica capillaries were applied for the enantiomeric separation of the ephedrine–pseudoephedrine (E-PE) and -methylphenethylamine (-Me-PEA) isomers by using CE method [173]. On the other hand, GO-polymer coated fused-silica capillary columns were used to improve the enantiomeric separation of three anionic racemic drugs (naproxen, warfarin and pranoprofen) by using CEC with methyl-β-cyclodextrin (Me-β-CD) as chiral selector [107]. Using molecular modeling with AutoDock, the group studied the mechanism of GO-modification effect on the enantioseparation efficiency of the CEC system. In another work, Hong et al. [174] reported for the first time the development of GO-based affinity capillary silica monolith with HSA or pepsin as chiral selector for enantiomeric separation and proteolysis by using CEC. It was observed that as compared to affinity monoliths without GO, HSA-modified affinity capillary silica monoliths (HSA-GO-EDA@CSM) possess better enantiorecognition ability. Recently, Li et al. [110] reported better HPLC enantiomeric separation of benzene-enriched enantiomers by using a novel CSP based on GO. In this method, GO was first covalently coupled to silica gel microspheres which was subsequently reduced with hydrazine to form reduced graphene oxide@silica gel (rGO@SiO_2_). Finally, the surfaces of rGO@SiO_2_ were physically coated with cellulose tris-(3,5-dimethylphenylcarbamate) to prepare the CSP. Most recently, a smart multifunctional graphene oxide nanocomposite was prepared which showed exceptionally good selectivity, thermosensitivity and magnetic separability for the identification and enantiomeric separation of Trp enantiomers [111]. The synthesized nanocomposite was composed of GO nanosheets, immobilized superparamagnetic Fe_3_O_4_ nanoparticles, poly(*N*-isopropylacrylamide-co-glycidyl methacrylate) (PNG) chains and β-CD which was fabricated through a combination of surface-initiated atom transfer radical polymerization (SI-ATRP) and a ring-opening reaction, as shown in Figure 10.

## 4. Conclusions

Enantiomeric recognition and separation is one of the most challenging tasks, particularly in the fields of contemporary pharmaceutical science, agrochemical science, material science, and many other rapidly expanding areas of research. Notably, the advent of modern high-throughput experimentation (HTE) technology has enabled the research and industrial laboratories to produce a large number of samples within a very short period of time. In comparison, the analytical methods (e.g., HPLC, GC, CE, etc.) which are generally employed for screening the reaction yield, enantiomeric purity, stability to racemization, enantiomeric excess (ee), and concentration of chiral compounds are relatively expensive, time consuming, laborious and produces solvent waste [175,176,177]. In this context, chiral sensing methods, capable of performing real time analysis of mixtures of enantiomers by utilizing inexpensive instrumentation with almost zero waste of expensive reagents, have significant scientific and industrial relevance and, therefore, are gaining increasing attention [177]. To date, a number of methods including electrochemical sensors, gravimetric-mass sensors-resonators, electrical sensors and chiroptical/spectroscopic sensors have been developed for fast and accurate differentiation of enantiomer [178]. Table 3 summarizes some of the recently used chiral sensing methods. Importantly, there are also examples where NPs have been used for the purpose of chiral sensing. For example, Tsourkas et al. [179] reported a enantioselective immunosensor by utilizing dextran-coated MNPs as magnetic relaxation switch that decreased the T2 relaxation time of water by 100 ms. The group successfully detected 0.1 μM of d-Phe in the presence of 10 mM of l-Phe (99.998% ee) by using NMR measurement of the T2 parameter. In another work, Wang et al. [58] reported a visual sensing platform based on AuNPs for visual recognition of Gln enantiomers. Silver nanoparticles (~400 nm) have been used to enhance the circular dichroism by two orders of magnitude [180]. Moreover, enantioselective sensors based on microcantilevers (MCs) with nanostructured (roughened) gold surfaces on one side showed promising results for the label-free stereoselective detection of α-amino acids [181]. Further, use of molecular imprinted polymer (MIP) nanowires/nanofibers showed immense potential to enhance the sensitivity of chiral sensors by allowing more binding sites (or cavities) due to the high surface area of such nanostructures [182,183]. Thus, integration of nanoscience and nanotechnology has a lot of potential to enhance the molecular sensing and signal transduction, which are the basic processes of chiral sensing [184]. A more detailed analysis of the chiral sensing methods can be found in several recent reviews [177,184,185] and are not discussed here.

In this review, we mainly focus on the application of nanostructured materials to amplify chiral recognition and separation. Notably, a wide array of nanostructures get benefited from the rapid development of nanotechnology are synthesized for the characterization and purification of racemates. The surfaces of NPs coated with chiral ligands (chiral NPs) have proved the potentialities for enantiomeric recognition based on the specific interactions between analytes and target enantiomers. Chiral NPs have paved a new route for enantiomeric recognition and surpassed the current cumbersome methods in this field, offering a relatively simple examination platform as discussed in Section 2. Notably, chiral NPs accommodate various nanocomposites to manipulate the molecular adsorption and aggregation, colloidal assembly, and to characterize chemical dynamics at particle surfaces as a useful modality for the understanding of enantioselective mechanism and future pharmaceutical applications. These surface effects also raise questions concerning the means by which biologically active compounds interact with chiral molecules, particularly with regard to enantioselective mechanisms at the nanoscale.

On the other hand, separation of racemic compounds is accomplished by selecting the desired enantiomer by introducing chiral selectors of compatible size and structure to the separation system as a part of the CSP or as chiral additive in the mobile phases (CAMP) [9,204]. Notably, there still remain three major issues on the use of chiral selectors for the detection and/or separation of enantiomers. These are: (a) the broad spectrum of applications, (b) the cost of the selector and (c) the productivity of the separation (gram of pure enantiomers/kg of CSP/day). In this regard, it is noteworthy to mention that α-, β- and γ-CDs (oligosaccharides) and their derivatives seem to represent a kind of universal chiral selector not only due to their broad-spectrum of chiral selectivity and relatively low cost but also their applicability in most of the separation techniques, such as, TLC, CEC, HPLC, CE, crystallization, etc. In addition, modified polysaccharides, proline derivatives, Pirkle type chiral selectors are also finding widespread applications in both analytical and preparative scale separations [9,205,206]. Recently, the most important and widely used CSPs for enantio-recognition/separation mechanisms were discussed in several reviews [9,21,207,208,209,210,211]. Importantly, economics of the separation process including the selection of the CSP/CAMP and the separation technique depends mainly on the stage of production. For example, in early stage small scale laboratory productions, finding an optimum separation method which is capable of producing large amount of desired enantiomers within a suitable time period becomes more important than the cost of the process. For preparative scale productions, however, both cost of the process as well as its productivity plays an important role [212,213]. Importantly, during the transition from analytical to preparative scale productions, conventional separation techniques (e.g., batch chromatography) are often replaced by high throughput large scale production techniques like stimulated moving bed (SMB) technology [214]. Further, in preparative scale separations the cost of production may significantly vary depending on the type of CSP/CAMP used and the technique in which the separation process is conducted. Thus the primary challenge is to find an optimum (cost effective and most productive) separation process, especially by using all possible combinations of more efficient CSPs/CAMPs [213,215]. In this regard, the use of nanoparticles and nanocomposites in separation processes has the potential to circumvent such challenges. In Section 3, we described the state of the art in the application of nanostructured materials in the field of enantiomeric separation. Arguably the nanostructured materials will continue to play a vital role in the separation of chiral molecules, especially due to their extraordinary capacity to enhance the enantioseparation ability of conventional techniques.

To summarize, we note that enantioseparation science has completed a phenomenal journey of around 170 years since its first demonstration in 1848 by Louis Pasteur [216]. At the present time, combination of conventional enantioseparation techniques with the recent advances in nanoscience and nanotechnology is proving to be quite synergetic by producing exciting results. Despite all these developments, the major challenge that still remains is the development of a more flexible, efficient and cost-effective chiral resolution technique and/or chiral selector to efficiently separate the numerous newer and newer chiral compounds, especially chiral drugs that are continuously being introduced. Nevertheless, without forgetting the enormous research efforts being devoted in this area during the last few years, we anticipate that the near future shall witness smarter techniques with more universal abilities for enantiomeric sensing, recognition, and separation.

## Figures and Tables

**Table 1:** Comparison of currently existing chiral resolution methods. The table and the caption have been adapted with permission from [50], Copyright © 2008 The Royal Society of Chemistry. Note that the original Table is slightly modified and the method “Self-disproportionation of enantiomers” has been added.

Methods	Advantages	Disadvantages	Possible Scale
(a) Crystallization resolution			
(a1) Direct or preferential crystallization	Simplicity, low cost	Batch operation, resolving conglomerate	Small- and large-scale
(a2) Diastereomeric crystallization	Simplicity, wide applicability	Expensive, difficulty in finding appropriate resolving agents	Large-scale, industrial scale
(b) Kinetic resolution			
(b1) Chemical-mediated	High stability	Low efficiency	Preparative scale, large-scale
(b2) Enzyme-mediated	High resolving efficiency	Decreasing enzyme activity, narrow application range	Preparative scale, large-scale
(c) Chromatographic separation			
(c1) Supercritical fluid chromatography	Lower costs, *^a^* high efficiency, resolving most racemates	Low capacity,	Large-scale
(c2) Simulated moving bed chromatography	Continuous operation, *^a^* high efficiency, resolving most racemates	Low capacity,	Large-scale
(c3) Other chromatography	High efficiency, resolving most racemates	Low capacity, expensive, batch operation, slow and labor intensive	Analytical scale, preparative scale
(d) Membrane-based separation	Low cost, energy saving, high capacity, continuous operation and easy scale-up	Low number of transfer units per apparatus	Large-scale, industrial scale
(e) Self-disproportionation of enantiomers	Ubiquitous and spontaneous, simple, cost effective, and fully predictable (SDE via centrifugation), can be used for both liquid and crystalline Compounds (SDE via chromatography), all forms of liquid chromatography have the potential to give rise to SDE [44,48]	Does not occur with racemic compounds—instead it occurs only in case of partly enriched chiral compounds [51]	Analytical scale, preparative scale [44]

*^a^* Note: Advantages of (c1) and (c2) were obtained by comparing with high performance liquid chromatography.

**Table 2:** Some representative examples in which nanomaterials have been used for the separation of chiral compounds [24,85,86].

Type of Nanostructured Material		Method of Separation/Characterization	Chiral Selector/Template	Nanostructure Dimensions	Analytes/Analysed Compounds	Ref.
Metallic nanoparticles	AgNP	CE	β-CD	AgNPs were of the size of ca. 21 nm	1-phenyl-1-propanol, 1-phenyl-2-propanol, and 2-phenyl-1-propanol	[87]
		Colorimetry	Nucleotide-capped AgNPs	-	d,l-Cys	[52]
	AuNP	Optical polarimetry	d,l-Cys-AuNPs	Average diameter of AuNPs: ~5 nm	Propylene oxide	[88]
		Colorimetry	*N*-acetyl-l-Cys-capped AuNPs as chiral candidate	Size range from 6 to 8 nm	d,l-Tyr	[53]
		Optical polarimetry	Tetrahexahedral (THH, 24-sided) AuNPs modified with d- or l-Cys was used as chiral separator	Shape: rod-like; diameter: ~40 nm; length: ~100 nm	Propranolol	[89]
		CE	Streptomycin-modified gold nanoparticles (ST-AuNPs)	Particle size of AuNPs and ST-AuNPs was 53.1 nm and 79.2 nm, respectively	Adrenergic compounds: adrenaline, noradrenaline and isoprenaline	[86]
		Centrifugation	Functional nucleic acids-modified AuNPs	Diameter of AuNPs (for best separation efficiency): 55nm	d,l-Trp	[69]
		Pseudostationary phase-CEC	Thiolated β-CD-modified AuNPs	Average diameter: 9.5 ± 2.5 nm	Four amino acid enantiomers (d,l-Val, Leu, Glu and Asp) and three drug enantiomers (*R,S*-chlorpheniramine, zopiclone and carvedilol)	[90]
Metal Oxide nanoparticles	Iron Oxide (Fe_3_O_4_)	Direct separation using a magnet	(*R*)- and (*S*)-*N*-(2,2-dimethyl-4-pentanoyl)-proline-3,5-dimethylanilide	The average particle size of magnetic silica nanoparticles (MSNPs): 300 nm	*N*-(3,5-dinitrobenzoyl)-α-amino acid *N*-propylamides	[91]
		HPLC	Bovine serum albumin (BSA)	Mean diameter of Fe_3_O_4_: 400 nm; thickness of silica layer in Fe_3_O_4_@SiO_2_: 60 nm	Trp, Phe and His	[66]
		HPLC	Carboxymethyl-β-CD	-	d,l-Trp, Phe and Tyr	[92]
		HPLC	BSA	Average size: 13.3 nm	Ibuprofen and ofloxacin	[93]
	Titanium dioxide (TiO_2_)	HPLC	Cellulose tris-(3,5-dimethyl-phenylcarbamate)-coated TiO_2_/SiO_2_ chiral stationary phase (CSP)	Size of TiO_2_/SiO_2_ spheres: ~6 nm; pore diameter: ~7 nm	Eight basic indole ring derivative enantiomers	[94]
		CE	Tris-H_3_PO_4_ solution containing TiO_2_ NPs as background electrolytes (BGEs)	-	β-adrenergic drugs (atenolol, eliprolol, clorprenaline, fenoterol, metoprolol, propranolol, and terbutaline) and clenbuterol	[95]
	Zirconiun dioxide (ZrO_2_)	Separation using a magnet	Cellulose tris-(3,5-dimethylphenylcarbamate)	Average size: 340 nm	Basic β-blocker (β-antagonists) chiral drugs	[96]
Carbon nanostructures	Single-walled nanotubes (SWCNTs), multi-walled nanotubes (MWCNTs)	Electrokinetic chromatography (EKC)	SWCNTs and MWCNTs	SWCNT: diameters between 0.7 and 1.2 nm and lengths 2–20 mm; MWCNT: diameters between 6 and 20 nm and 1–5 mm length	(±)-ephedrine, (±)-norephedrine and (±)-*N*-methylephedrine	[97]
	SWCNTs	Microchip electrophoresis	BSA conjugated with the shortened carboxylic SWCNTs	-	Trp	[98]
		HPLC	CNT monolithic column coated with a pyrenyl derivative	Average diameter: 1 nm; length: < 10 nm	A series of 10 amino acids	[99]
		HPLC	SWCNTs in monolithic backbones	Average diameter: ~1 nm; length 1–10 μm	α- and β-blockers, antiinflammatory drugs, antifungal drugs, dopamine antagonists, norepinephrine-dopamine reuptake inhibitors, catecholamines, sedative hypnotics, diuretics, antihistaminics, anticancer drugs, and antiarrhythmic drugs	[100]
	MWCNTs	CE	β-CD	Interlayerspacing of 3.4 Å; typical diameter of 10–20 nm	Clenbuterol	[101]
		TLC	Hydroxypropyl-β-CD	Diameter: 10–20 nm; length: 2–20 µm	Clenbuterol	[102]
	Ionic liquid dispersed MWCNTs	EC	Chondroitin sulfate E	MWCNT (od: 10–20 nm, length 5–30 nm)	Racemic drugs (amlodopine, laudanosine, nefopam, citalopram, and propranolol)	[103]
	Carboxylated SWCNTs and MWCNTs	EKC	β-CD	Carboxylated SWCNTs: od 1~2 nm; Carboxylated MWCNTs: od 10~20 nm	Sulconazole, ketoconazole, citalopram hydrobromide, and nefopam hydrochloride	[104]
	Graphene	HPLC	Graphene nanosheets with tetracyanoethyle oxide (TCNEO) and (*S*)-(+)-2-pyrrolidinemethanol	-	Ibuprofen and thalidomide racemic mixtures	[105]
		TLC	d-TA-graphene	Thickness of graphene nanosheet: 2–3 nm	Racemic drugs (propranolol and ofloxacin)	[106]
	Graphene oxide (GO)	CEC	Methyl-β-CD	-	Anionic racemic drugs (naproxen,warfarin and pranoprofen)	[107]
		CEC	β-CD conjugated GO-magnetic nanocomposites (GO/Fe_3_O_4_ NCs)	Average size of about 8 nm	d,l-Trp	[108]
		Open-tubular capillary electrochromatography (OTCEC)	Bovine serum albumin-conjugated graphene oxide–magnetic nanocomposites GO/Fe_3_O_4_/BSA	-	Trp, threonine (Thr), and propranolol enantiomers	[109]
		HPLC	Reduced graphene oxide/silica gel (rGO/SiO_2_)	Silica gel (particle size of 5 μm, pore size of 120 Å)	Benzene enriched enantiomers, ibuprofen, *trans*-stilbene oxide, 2-phenylcyclohexanone, praziquantel, propranolol, *R*,*S*-equol, ketoconazole, benzoin, and quinidine	[110]
		HPLC	Graphene oxide/poly(*N*-isopropyl-acrylamide-co-glycidyl methacrylate) (MGO/PNG-CD)	Diameter: ~80 nm; thickness: 8nm	d,l-Trp	[111]
Other nanoparticles	Polystyrene nanoparticles	CE	Hydroxypropyl (HP)-β-CD	Average diameter: 15 ± 5 nm	Propranolol	[112]
		Chromatographic technique	Sulfated β-CD	Average size of ethylene dimethacrylate-*N*-methacryloyl-l-His methyl ester NP: ~111.5 nm	Ofloxacin	[113]
	Mesoporous silica nanoparticles	Direct chiral separation, CE	Teicoplanin-conjugated mesoporous silica MNPs	Average diameter: ~600 nm; mean pore size: ~3.9 nm	d,l-Trp, Phe, d,l-Mandelic acid, (±)-1-Phenyl-1,2-ethanediol, and *N*-Benzoyl-d,l-alanine	[67]
		CEC	Cellulose tris-(3,5-dimethylphenyl-carbamate)	Particle size of ca. 600 nm and a pore size of ca. 3 nm	Tetrahydropalmatine and pindolol	[114]
		CEC	Pepsin		(±)-nefopam	[115]
		CE	Carboxymethyl-β-CD	Approximately 120 nm	Ephedrine and chlorpheniramine	[116]
		CE	BSA	Approximately 150 nm	Propranolol and Trp	[117]
	Metal-organic framework	HPLC	Chiral bridging ligand	-	2-butanol and 2-methyl-1-butanol HPLC	[118]
		GC	Chiral bridging ligand	-	Amino acid derivative	[119]

**Table 3:** Representative list of chiral sensing methods. The table derives from Manoli et al. [177].

Sensors		Sub-Categories/Types	Advantages	Disadvantages	Related References
(a)	Electrochemical Sensors	Potentiometric sensors, voltammetric sensors	High sensitivity, simple operation, rapid detection, low cost, miniature size, low power requirements [186]	Poor durability, need for a reference electrode [177]	[187,188,189,190]
(b)	Gravimetric-Mass Sensors	Quartz crystal microbalance (QCM) devices based on: CDs, molecular imprinted polymers, biological recognition elements, etc.	Capability to measure sub-nanogram level changes, possibility of real-time condensed phase measurements, long time stability	Resolution degradation due to multi resonance modes of the cantilever, limited performance due to degraded quality factor and resolution in liquid medium [191], electrochemical QCM can only be used for studying electroplated, evaporated, or sputtered materials [185]	[192,193,194]
(c)	Electrical Sensors	Chemiresistors, organic field effect transistors, chemocapacitors	Ease of fabrication and simplicity in instrumentation, cost effective, large selection of materials and flexible	Low Thermal stability and low chemical stability (oxidation)	[195,196,197,198]
(d)	Optical sensors	SPR sensors, fluorescence spectroscopy, circular dichroism/optical rotation probes	Speed of detection, simplicity in the measurement procedure	Low sensitivity and poor tolerance to impurities [58,199]	[176,180,200,201,202,203]
